# Pasteurella canis/oralis: A Tale of the Immunocompromised

**DOI:** 10.7759/cureus.64227

**Published:** 2024-07-10

**Authors:** Brittany L Davis, Mark Rasnake

**Affiliations:** 1 Graduate Medical Education, Naples Community Hospital Healthcare System, Naples, USA; 2 Graduate Medical Education/Infectious Disease, Naples Community Hospital Healthcare System, Naples, USA

**Keywords:** transplant, surgery, internal medicine, septic shock, infectious disease

## Abstract

Bacteria from the *Pasteurella* genus are small Gram-negative coccobacilli. They are commonly found in the oral cavity, gastrointestinal tract, and upper respiratory tract of domestic pets. When humans experience trauma such as scratches, bites, or direct wound licking from an animal, this could result in skin or soft tissue infections. The purpose of this case report is to highlight that immunocompromised individuals are at greater risk of overwhelming sepsis and infections with the *Pasteurella* species. Moreover, it reemphasizes the importance of comprehensive questioning regarding exposures to any wild or domesticated animals as infectious sources in the setting of overwhelming sepsis in the immunocompromised.

## Introduction

The case report by Ramiro et al. [1] indicates that *Pasteurella *species are responsible for about 50% of wound infections from dog bites and around 75% from cat bites. *Pasteurella *is a gram-negative, non-spore-forming, and nonmotile coccobacillus that is penicillin-sensitive. These species are well-known for causing bacterial zoonotic infections in humans, particularly after animal bites, scratches, or the licking of open wounds, often leading to skin and soft tissue infections. Immunocompromised individuals face a higher risk of morbidity and mortality from *Pasteurella *infections, with risk factors including immunosuppressive therapy, diabetes, malignant tumors, liver failure, and animal exposure. We present the case of a 74-year-old immunosuppressed male who developed necrotizing fasciitis and septic shock secondary to *P. canis*/*oralis *after being scratched by his pet chihuahua.

## Case presentation

A 74-year-old male with a history of renal transplantation (dead donor kidney transplant) chronically on tacrolimus and prednisone, cirrhosis secondary to chronic alcohol use, and type 2 diabetes presented with altered mental status, fever, chills, diarrhea, generalized weakness, and fatigue three days after an abdominal paracentesis (5.8 L removed).

Vitals at presentation showed a temperature of 100°F, pulse of 69 bmp, respiratory rate of 16/min, and blood pressure of 89/40 mmHg (Table 1). On exam, he appeared chronically ill and toxic appearing with fluctuating mentation. Trace edema was noted in bilateral lower extremities, along with chronic bilateral venous stasis. Initial labs revealed lactic acid 4.7 mmol/L, creatinine 2.3 mg/dL, and white cell count 18.7x103 /mL (Table 2).

**Table 1:** Vitals *F: degrees Fahrenheit; pulse (bpm): pulse in beats per minute; respiratory rate (bpm): respiratory rate in breaths per minute; mmHg: millimeters of mercury

Vitals	Reference	Patient
Temperature (*F)	97.7–199.5*F	100
Pulse (bpm)	60–100	69
Respiratory rate (bpm)	12–20	16
Blood pressure (mmHg)	120/80	84/40

**Table 2:** Lab results

Labs	Reference	Patient
White blood cell count (10^3uL)	4.2–10.8	18.7
Lactic acid (mmol/L)	0.4–2	4.7
Creatinine (mg/dL)	0.6–1.3	2.3

**Figure 1:**
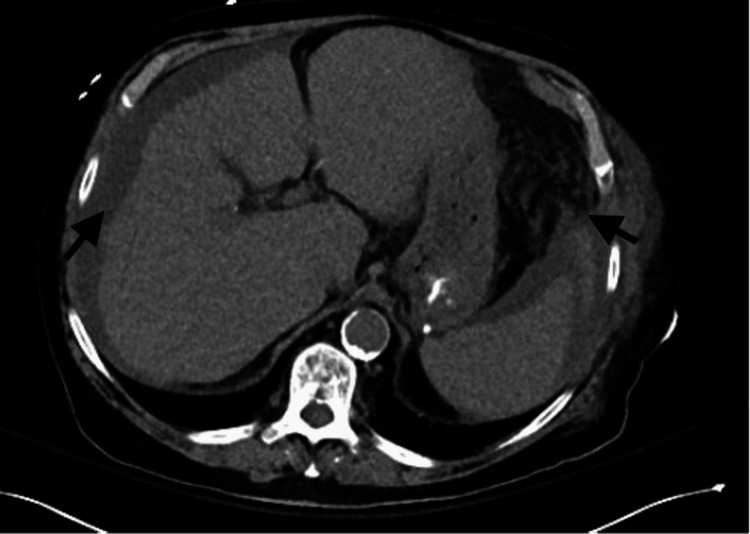
CT abdomen and pelvis: mild ascites indicated by black arrow (right). Left-sided flank fat stranding indicated by black arrow (left)

**Figure 2:**
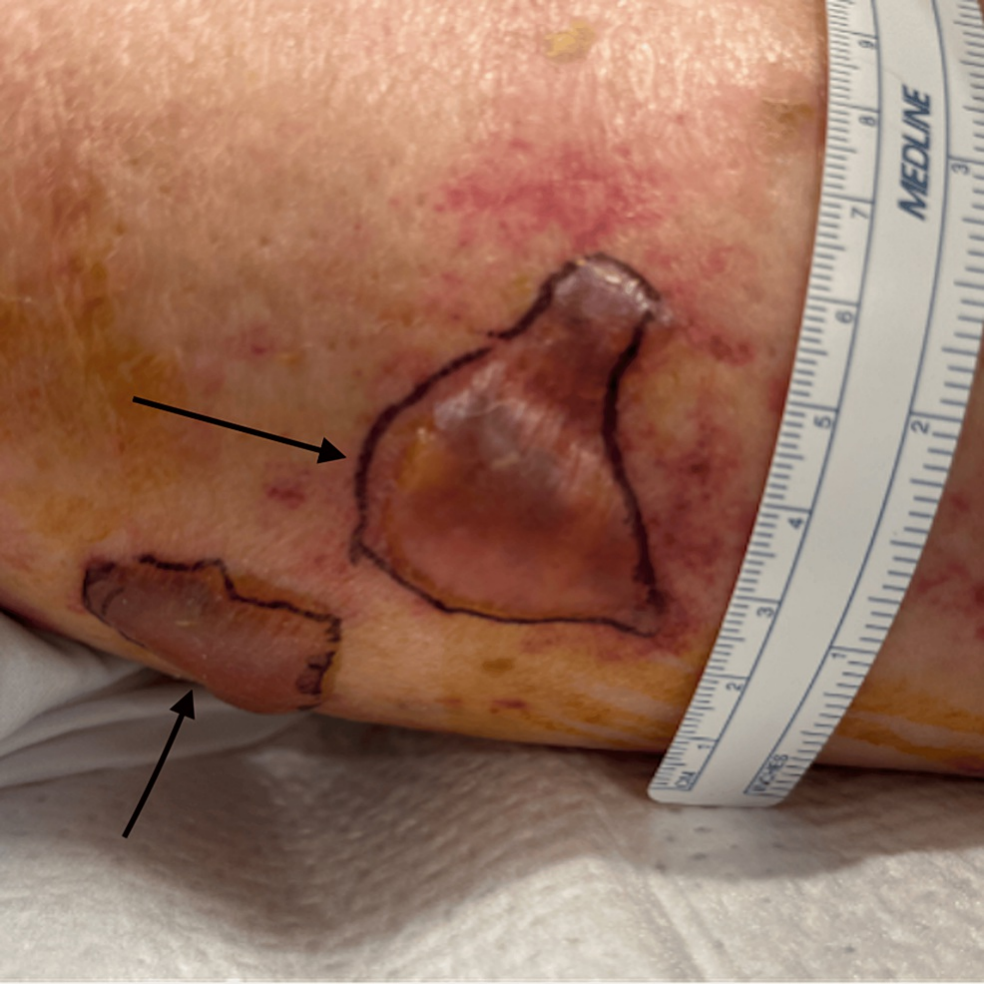
Left lateral-posterior thigh, tense purple bullae as indicated by black arrows

**Figure 3:**
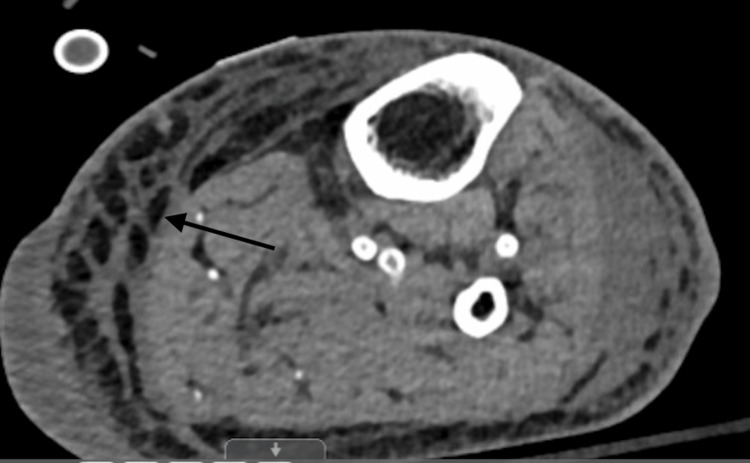
CT tibia and fibula left leg: diffuse soft tissue swelling as evidenced by black arrow

Chest radiography was negative for infiltrates, and CT of the abdomen revealed a cirrhotic liver with mild ascites and left flank fat stranding (Figure 1). He was initially given 2 L IV crystalloids and IV vancomycin and cefepime for sepsis without a clear source. His sepsis worsened, and he required three agents for vasopressor support, antibiotics were switched to levofloxacin, meropenem, and clindamycin. Shortly after admission, he developed worsening left lower extremity erythema accompanied by multiple tense and fluctuant purple bullae (Figure 2). CT tibia and fibula were ordered (Figure 3). Results were remarkable for diffuse soft tissue swelling throughout the entire leg. General surgery was consulted. Necrotizing fasciitis was diagnosed, and he underwent wide debridement of the left lower leg and a four-compartment fasciotomy. Blood and tissue cultures recovered *P. canis*/*oralis*, and antibiotics were adjusted to organism and susceptibilities.

Upon further discussion with the patient’s wife, she confirmed that her husband recently sustained trivial, superficial scratches on his anterior left shin from their pet chihuahua. No additional history of travel, past incarcerations, or sick contacts were noted.

Despite fluid resuscitation, antibiotic therapy, and surgical debridement, he continued to decline, suffered multifocal cerebral strokes, and ultimately died on hospital day 13.

## Discussion

Infections with *Pasteurella *spp. usually occur because of cat scratches or dog bites. They may also occur if an open wound or sore is licked by an animal. In immunocompromised individuals, the wound may be quite trivial in appearance, as was the case in our patient, and such contact might not be brought to the attention of medical staff. A thorough exposure history must be sought when immunocompromised patients present with severe sepsis. In such patients' infections with *Pasteurella *spp. carry substantial morbidity and mortality and require prompt antibiotic therapy and surgical source control. Several virulence factors of the *Pasteurella* spp. allow them to cause overwhelming infections. As highlighted by Peng et al. [2]. Capsules allow evasion of the immune system; lipopolysaccharides are a well-known virulence factor of gram-negative bacteria, and hyaluronidase further promotes the virulence factors by disseminating them throughout the host tissue. It causes tissue inflammation, which subsequently leads to the release of pro-inflammatory cells. In this case, our patient was promptly treated with broad-spectrum antibiotics. He also underwent an urgent fasciotomy with general surgery because of necrotizing fasciitis. Prophylaxis or active local infections may be treated with amoxicillin-clavulanate [3]. In the setting of overwhelming sepsis ampicillin-sulbactam, piperacillin-tazobactam or a carbapenem may be used. In this case, the patient presented with overwhelming sepsis and was covered with vancomycin and cefepime. He was later transitioned to meropenem.

As mentioned in the case report written by Maraki et al. [4], bacteria from the *Pasteurella *genus are small Gram-negative coccobacilli. They are commonly found in the oral cavity, gastrointestinal tract, and upper respiratory tract of domestic pets. This study also demonstrated *Pasteurella *spp. accounts for approximately 50% of wound infections from dog bites and approximately 75% from cat bites.

Our case presentation further demonstrates that when humans experience trauma such as scratches, bites, or direct wound licking from an animal, this could result in overwhelming skin or soft tissue infections complicated by severe multisystem organ failure. The case report by Razali et al. [5] supports the fact that animals carry multiple zoonotic bacterial strains in their mouth, which include *Pasteurella *spp.

This case report aims to highlight that immunocompromised individuals are at greater risk of overwhelming sepsis and infections with bacteria from *Pasteurella *spp. Moreover, it reemphasizes the importance of comprehensive questioning regarding exposures to any wild or domesticated animals as infectious sources.

Similar to the case report by Boadu et al. [6], an immunocompromised patient developed a refractory infection and was later noted to have *Pasteurella *bacteremia secondary to a cat scratch. Similar to our case presentation, further questioning revealed that he was scratched by his pet chihuahua. The case also highlights that clinicians must have a high clinical index of suspicion when caring for immunocompromised patients presenting with severe septic shock.

## Conclusions

Immunocompromised patients presenting with severe overwhelming sepsis should have a thorough exposure history taken. This is important in targeting and focusing antibiotic therapy and planning for early surgical intervention.
